# Correction to ‘8-Oxoguanine incorporation into DNA repeats in vitro and mismatch recognition by MutSα’

**DOI:** 10.1093/nar/gkaf374

**Published:** 2025-05-06

This is a correction to: Peter Macpherson, Flavia Barone, Giovanni Maga, Filomena Mazzei, Peter Karran, Margherita Bignami, 8-Oxoguanine incorporation into DNA repeats *in vitro* and mismatch recognition by MutSα, *Nucleic Acids Research*, Volume 33, Issue 16, 1 September 2005, Pages 5094–5105, https://doi.org/10.1093/nar/gki813

In February 2025, the Editors were made aware of a potential splice line between lanes 10 and 11 in the Western-Blot image presented in Fig. 3B.

The authors no longer have the original data but clarified that the image originated from a single experiment. Due to the gels' capacity to run only 10 samples per gel, the original image comprises two blots: one with 10 lanes on the left and another with 6 lanes on the right. Four empty lanes were removed from the second blot.

A revised Fig. 3B, now featuring a line to distinguish the two blots, is provided below.


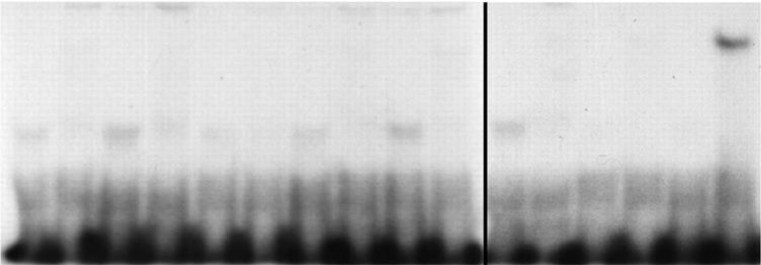


These details have been corrected only in this **correction notice** to preserve the published version of record.

